# Longitudinal changes in intracortical excitability during Ramadan diurnal intermittent fasting: A paired-pulse transcranial magnetic stimulation study

**DOI:** 10.1371/journal.pone.0349740

**Published:** 2026-08-31

**Authors:** Meeyoung Kim, Ibrahim M. Mostafa, Alham Al-Sharman, Nabil Saad, Hanan Khalil, Hikmat Hadoush

**Affiliations:** 1 Department of Physiotherapy, College of Health Sciences, University of Sharjah, Sharjah, United Arab Emirates; 2 Laboratory of Health Science & Nanophysiotherapy, Department of Physical Therapy, Graduate School, Yongin University, Yongin, Republic of Korea; 3 Neuromusculoskeletal Rehabilitation Research Group, Research Institute of Medical and Health Sciences, University of Sharjah, Sharjah, United Arab Emirates; 4 Department of Rehabilitation Sciences, Faculty of Applied Medical Sciences, Jordan University of Science and Technology, Irbid, Jordan; 5 Department of Rehabilitation Sciences, College of Health Sciences, QU Health, Qatar University, Doha, Qatar; Applied Science University, PALESTINE, STATE OF

## Abstract

Ramadan diurnal intermittent fasting (RDIF) represents a natural model of prolonged daily intermittent fasting associated with metabolic and circadian alterations. This study investigated longitudinal changes in intracortical excitability across pre-, mid-, and post-Ramadan timepoints in healthy adults observing RDIF. Thirty fasting participants in this prospective observational cohort study underwent paired-pulse transcranial magnetic stimulation at three timepoints (pre-, mid-, and post-Ramadan). A non-fasting control group (n = 11) was assessed at pre- and mid-Ramadan. Conditioned motor-evoked potentials were recorded at interstimulus intervals of 2–10 ms and normalized to unconditioned responses. A linear mixed-effects model assessed effects of Timepoint and interstimulus interval (ISI). Secondary outcomes included blood glucose, cognitive performance, sleep duration, and reaction time. A significant main effect of Timepoint (p < 0.001) indicated longitudinal modulation of intracortical excitability, with increased MEP ratios at mid-Ramadan and partial persistence post-Ramadan. The ISI effect confirmed the inhibition–facilitation gradient (p < 0.001). The Timepoint × ISI interaction was not significant (p = 0.566), indicating a global shift in excitability without ISI-specific modulation. Blood glucose and sleep duration decreased significantly at mid-Ramadan. RDIF is associated with a time-dependent increase in intracortical excitability, most appropriately interpreted as a generalized shift rather than selective modulation of inhibitory or facilitatory circuits. These changes occur in the context of concurrent metabolic, sleep, and circadian alterations and may reflect combined physiological influences of these factors; however, their individual contributions cannot be disentangled within the present naturalistic observational design. Because the non-fasting comparison group was not evaluated after Ramadan, the persistence of elevated intracortical excitability beyond Ramadan requires confirmation in adequately controlled longitudinal studies.

## Introduction

Ramadan diurnal intermittent fasting (RDIF) constitutes a naturalistic model of prolonged daily intermittent fasting practiced by over one billion Muslims worldwide. During Ramadan, individuals abstain from food and fluid intake from dawn to sunset for approximately 29–30 days, resulting in daily fasting durations of 12–18 hours. This pattern induces predictable metabolic consequences including reduced circulating glucose, altered lipid metabolism, and shifts in insulin and glucagon secretion [1–3]. Concurrently, modifications in sleep architecture, meal timing, and circadian entrainment are well documented [4–6].

The central nervous system is highly sensitive to both metabolic and circadian variation. Glucose represents the primary fuel for neuronal activity, and reductions in its availability alter synaptic transmission and intracortical excitability through effects on GABAergic interneurons, which are particularly metabolically vulnerable [7–9]. Circadian phase shifts influence GABAergic and glutamatergic signaling, thereby modulating the principal mechanisms of intracortical inhibition and facilitation [10,11].

Paired-pulse TMS provides a validated, non-invasive method to assess intracortical inhibitory and facilitatory circuits in vivo. Short-interval intracortical inhibition (SICI), assessed at ISIs of 2–4 ms, is mediated predominantly by GAB*AA* receptor activity at cortical interneurons [12,13]. Intracortical facilitation (ICF), measured at ISIs of 8–10 ms, predominantly reflects glutamatergic excitatory interneuronal activity [13–15]. Changes in the MEP ratio—the conditioned MEP amplitude normalized to the unconditioned MEP—thus provide a sensitive index of shifts in the cortical inhibition–excitation balance.

Prior work on metabolic and sleep-related influences on cortical excitability has yielded inconsistent findings, in part due to cross-sectional designs and single time-point assessments [10,11]. Longitudinal TMS studies spanning Ramadan are lacking. Ramadan diurnal intermittent fasting is not an isolated metabolic manipulation but an integrated physiological state in which daytime abstention from food and fluid, circadian phase shifts, and reduced sleep duration occur together. The present study was designed to characterize how intracortical excitability changes across this state over time, tracking the fasting cohort within-subject across pre-, mid-, and post-Ramadan timepoints. We hypothesized that this integrated exposure would be associated with a generalized increase in intracortical excitability, most pronounced at mid-Ramadan and partially resolving thereafter, without assuming selective modulation of specific inhibitory or facilitatory circuits.

## Materials and methods

### Participants

**Fig 1:**
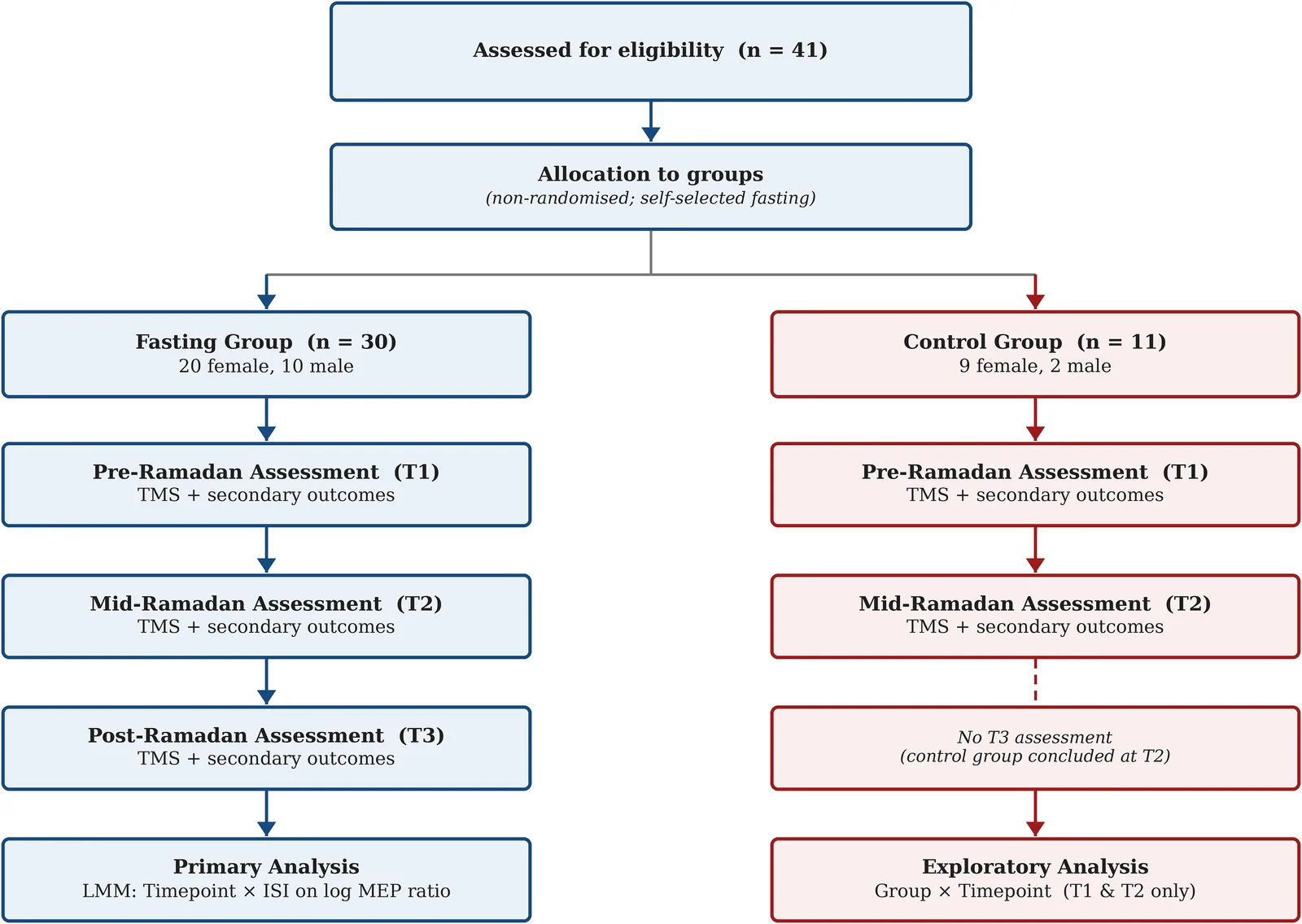
Participant flowchart. Forty-one participants were enrolled following screening. The fasting group (n = 30) was assessed at three timepoints; the control group (n = 11) was assessed at T1 and T2 only. Numbers available for the primary TMS outcome differed from enrolled totals due to missing or unusable MEP data at some ISIs.

Forty-one healthy young adults were recruited at the University of Sharjah, United Arab Emirates, where all assessments were performed: a fasting group (n = 30; 20 female, 10 male; mean age 19.9 ± 1.4 years; BMI 25.6 ± 6.0 kg/m²) and a non-fasting control group (n = 11; 9 female, 2 male; mean age 19.5 ± 0.9 years; BMI 23.7 ± 1.4 kg/m²). Demographic characteristics are presented in Table 1. Inclusion criteria comprised: age 18–35 years, no neurological or psychiatric disorder, no psychoactive or neuroactive medication, no history of epilepsy, right-hand dominance, and no TMS contraindications. Menstrual-cycle phase, contraceptive use, and hormonal status were not applied as inclusion or exclusion criteria, as the assessment schedule was fixed by the Ramadan calendar and could not be aligned to individual cycle phase. Groups did not differ significantly on any demographic variable at baseline (all *p* > 0.48; Table 1). A participant flowchart is presented in Fig 1.

**Table 1:** Participant demographic characteristics at baseline.

Variable	Fasting (n = 30)	Control (n = 11)	p-value
Age (years), mean ± SD	19.9 ± 1.4	19.5 ± 0.9	0.487
Sex (Female/ Male)	20/ 10	9/ 2	0.676^a^
Height (cm), mean ± SD	166.8 ± 9.6	165.7 ± 5.0	1.000
Weight (kg), mean ± SD	71.6 ± 19.7	65.3 ± 7.6	0.877
BMI (kg/m²), mean ± SD	25.6 ± 6.0	23.7 ± 1.4	0.758
Sleep (hrs/night), mean ± SD	7.5 ± 1.7	7.0 ± 0.7	—
Caffeine (cups/day), mean ± SD	0.6 ± 0.9	0.7 ± 0.5	—
Non-smokers, n (%)	21 (70%)	6 (55%)	—

Values are mean ± SD unless stated. p-values from Mann-Whitney U test unless noted. ^a^ Fisher’s exact test. — = not formally tested.

### Ethics statement

The study was conducted in accordance with the Declaration of Helsinki and was approved by the Research Ethics Unit, University of Sharjah (Reference: REC-25-01-30-01-PG; approved 9 October 2025). Written informed consent was obtained from all participants. Arabic-speaking participants provided consent using an approved Arabic-language consent form.

### Study design and timepoints

A longitudinal repeated-measures design was employed. The fasting group was assessed at T1 (pre-Ramadan, one week before Ramadan), T2 (mid-Ramadan, approximately days 14–16), and T3 (post-Ramadan, after the conclusion of the Eid al-Fitr holiday and within seven days of the final fasting day of Ramadan). The T3 window was defined in this way so that assessments fell after the acute behavioral perturbations of the Eid festivities had subsided, while remaining within the first week following the cessation of fasting. This scheduling approach is consistent with previous Ramadan studies that performed mid-Ramadan assessments during the second week of Ramadan [16]. In three female participants whose anticipated menstruation coincided with the scheduled T2 assessment, testing was performed on Ramadan day 12 to ensure assessment during active fasting.

The control group was assessed at T1 and T2 only. All sessions were conducted between 12:00 and 15:00 to minimize circadian variability in cortical excitability [10,11]. Data collection was conducted between 11 February and 26 March 2026. This observational cohort study is reported in accordance with the Strengthening the Reporting of Observational Studies in Epidemiology (STROBE) guideline [17]. A completed STROBE checklist is provided as Supporting Information (S1 Table).

### TMS protocol

Participants were seated in a high-back reclining chair with the head and neck supported and the right forearm and hand resting on a cushioned support to promote complete muscular relaxation. Continuous surface electromyography (EMG) was monitored in real time throughout the session to confirm resting muscle state, and stimulation was delivered only during periods of electrical quiescence in the target muscle. Surface EMG (TruTrace) electrodes were placed in a belly–tendon montage over the right first dorsal interosseous (FDI) muscle, with a ground electrode over the ipsilateral wrist. The FDI was selected as the target muscle for its well-characterized cortical representation within the primary motor cortex (M1) and its established use in paired-pulse TMS protocols [13].

Cortical excitability was assessed using a DuoMAG MP-Dual stimulator (Deymed Diagnostics, Hronov, Czech Republic) connected to a 70 mm figure-of-eight coil positioned over the hand area of the left M1. The coil was held tangential to the scalp with the handle oriented postero-laterally at approximately 45° to the mid-sagittal plane, thereby inducing a posterior-to-anterior current across the central sulcus [12]. The FDI motor hotspot was localized by systematically repositioning the coil in approximately 1 cm steps across the hand area of the left M1 and identifying the scalp site that elicited the largest and most consistent motor-evoked potentials (MEPs) in the contralateral FDI at the lowest stimulator intensity. This site was marked to maintain stable coil placement throughout the session, and all subsequent measurements were referenced to it.

Resting motor threshold (RMT) was determined at the identified hotspot and defined as the minimum stimulator output required to elicit MEPs of ≥50 μV peak-to-peak amplitude in at least 5 of 10 consecutive trials with the FDI at rest [12]. The conditioning stimulus (CS) was set at 80% RMT and the test stimulus (TS) at 120% RMT, consistent with standard paired-pulse protocols.

Paired-pulse TMS was administered at interstimulus intervals (ISIs) of 2, 4, 6, 8, and 10 ms, spanning the short-interval intracortical inhibition block and the rising phase of intracortical facilitation (ICF). At each ISI, the subthreshold CS (80% RMT) preceded the suprathreshold TS (120% RMT). Ten conditioned MEP trials were recorded per ISI, together with ten unconditioned TS trials for normalization. Peak-to-peak MEP amplitude and onset latency were measured for all trials. All measurements were conducted in the dominant hemisphere only, consistent with established protocols [13].

Group allocation was not randomized, as Ramadan fasting was a self-selected religious practice rather than an assigned intervention; participants and investigators were therefore not blinded to fasting status. This is an inherent feature of the naturalistic observational design. Within each session, the five interstimulus intervals were administered in a fixed ascending order (2, 4, 6, 8, and 10 ms) rather than in a randomized or interleaved sequence. Because randomized or pseudorandom interleaving of interstimulus intervals is often used in paired-pulse protocols, the fixed order used here may have introduced order- or time-related effects on the conditioned responses, which we acknowledge as a methodological limitation.

### Outcome measures

#### Primary outcome.

MEP ratio (conditioned/ unconditioned MEP amplitude) for each ISI. Ratios < 1.0 indicate SICI; ratios > 1.0 indicate ICF [13,18].

#### Secondary outcomes.

Blood glucose (mg/dL; finger-prick glucometry immediately pre-TMS) [8], image recall test [19], Stroop color-word task (response time and accuracy) [20], and simple reaction time (ruler-drop test, cm) [21].

### Statistical analysis

Conditioned MEP amplitudes were divided by the corresponding unconditioned MEP amplitude to yield MEP ratios. Distributions were non-normal (Shapiro-Wilk, all *p* < 0.001); a logarithmic transformation was applied prior to analysis.

A linear mixed-effects model (LMM) assessed fixed effects of Timepoint (3 levels) and ISI (5 levels) and their interaction on log-transformed MEP ratios, with participant as a random intercept. Model significance was evaluated using likelihood ratio tests (LRT) under maximum likelihood estimation. The model was estimated from all available observations under a missing-at-random assumption, without imputation.

Secondary outcomes were examined using Friedman tests, with Wilcoxon signed-rank post-hoc comparisons (Bonferroni-corrected α = 0.017). Between-group comparisons used the Mann-Whitney *U* test. Effect sizes are reported as rank-biserial *r*. All analyses were conducted in IBM SPSS Statistics (version 29.0; IBM Corp., Armonk, NY, USA). Significance was set at *p* < 0.05 (two-tailed).

### Statistical power and cohort design

This study was designed as a prospective, longitudinal, repeated-measures investigation of a single fasting cohort, with the within-subject linear mixed-effects model as the primary analysis. No a priori sample-size or power calculation was performed. Recruitment used convenience sampling: we enrolled the maximum number of eligible, compliant participants who could be assessed within the fixed calendar window of the 2026 Ramadan period, a constraint inherent to studying this naturally occurring exposure. The fasting cohort (n = 30) was assessed at all three timepoints, whereas the non-fasting cohort (n = 11) was smaller and was assessed at pre- and mid-Ramadan only. The study was therefore not powered to detect between-group differences or Group × Timepoint interactions, and all such analyses are reported as highly exploratory. Accordingly, the between-group component of this work is best regarded as an exploratory pilot comparison intended to provide contextual reference and to inform the design of adequately powered future trials, while the primary conclusions rest on the within-subject longitudinal findings in the fasting cohort. Between-group results should not be generalized beyond the present sample.

## Results

### Participant characteristics

Demographic data are in Table 1 and the participant flowchart is in Fig 1. The fasting group comprised 30 participants (20F/10M; age 19.9 ± 1.4 years; BMI 25.6 ± 6.0 kg/m²). The control group comprised 11 participants (9F/2M; age 19.5 ± 0.9 years; BMI 23.7 ± 1.4 kg/m²).

### Unconditioned MEP amplitude and latency

Unconditioned MEP data are presented in Table 2. MEP amplitude did not change significantly across timepoints in the fasting group (Friedman χ² = 5.08, df = 2, *p* = 0.079). The T1–T3 pairwise comparison was also non-significant after correction, although the associated effect size was moderate (Wilcoxon *W* = 86, *p* = 0.069, *r* = 0.43). MEP latency was stable across all three timepoints (Friedman χ² = 2.04, df = 2, *p* = 0.360). No significant between-group differences were observed at T1 or T2 (all Mann-Whitney *p* > 0.05).

**Table 2:** Unconditioned MEP amplitude (µV) and onset latency (ms) by group and timepoint (fasting group; n = 30, control group; n = 11).

Measure	Group	Pre-Ramadan (T1)	Mid-Ramadan (T2)	Post-Ramadan (T3)	p	r
MEP amplitude (µV)	Fasting	1124 [764–1705]	1194 [621–1906]	691 [417–2007]	0.079	*0.43*
	Control	746 [437–1055]	457 [281–825]	—	—	*—*
MEP latency (ms)	Fasting	22.7 [21.6–23.7]	22.8 [21.1–24.2]	21.9 [21.6–23.6]	0.360	*—*
	Control	22.2 [21.3–23.1]	21.9 [21.4–22.7]	—	—	*—*

Values are median [IQR]. Sample size varied by measure and timepoint due to available-case analysis. — = not assessed. p from Friedman test; r = rank-biserial correlation (Wilcoxon signed-rank, T1–T3 comparison in fasting group).

### Cortical excitability: Paired-pulse TMS

**Fig 2:**
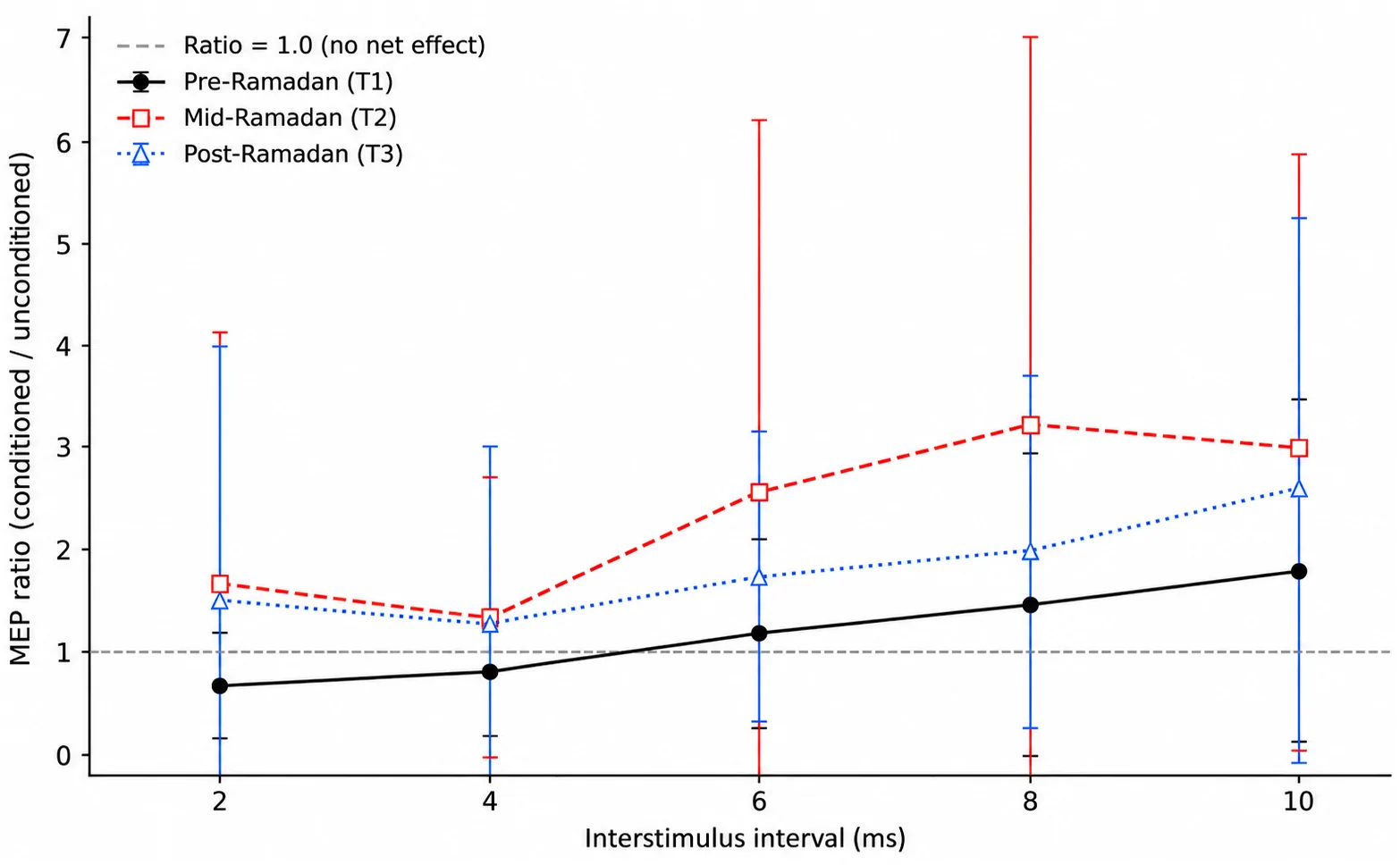
Mean conditioned MEP ratios (± SD) as a function of interstimulus interval (ISI) at each assessment timepoint in the fasting group. MEP ratio = conditioned MEP amplitude/ unconditioned MEP amplitude. Values below 1.0 indicate short-interval intracortical inhibition (SICI); values above 1.0 indicate intracortical facilitation (ICF). The horizontal dashed reference line indicates a ratio of 1.0 (no net effect). A significant main effect of Timepoint was observed (χ² = 14.53, df = 2, p < 0.001); the Timepoint × ISI interaction was not significant (p = 0.566).

**Fig 3:**
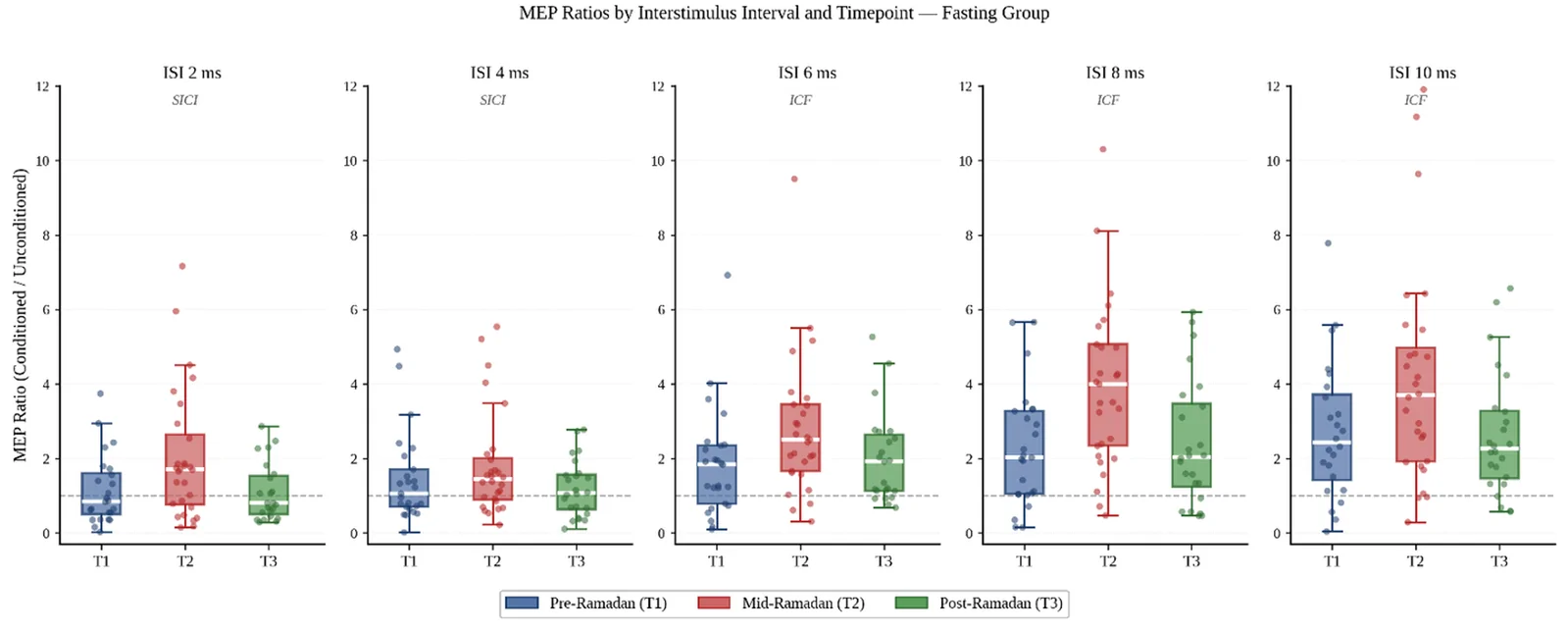
Distribution of conditioned MEP ratios at each interstimulus interval (ISI) across timepoints in the fasting group. Box plots show median (horizontal line) and interquartile range; whiskers extend to 1.5 × IQR; values beyond whiskers are not plotted. Individual data points are overlaid with random horizontal jitter for visibility. The horizontal dashed reference line indicates a ratio of 1.0 (no net inhibition or facilitation). Ratios below 1.0 indicate short-interval intracortical inhibition (SICI); ratios above 1.0 indicate intracortical facilitation (ICF). T1 = pre-Ramadan; T2 = mid-Ramadan; T3 = post-Ramadan.

MEP ratio descriptive statistics are in Table 3; LMM results are in Table 4; Fig 2 illustrates the ISI profiles by timepoint. In the fasting group, MEP ratios followed a consistent pattern across all ISIs: lowest at T1, highest at T2, and partially attenuated at T3. The distribution of individual MEP ratios across timepoints is further illustrated in Fig 3. At ISI 2 ms, the mean ratio increased from 0.68 ± 0.51 at T1 to 1.68 ± 2.43 at T2. At ISI 10 ms (ICF), ratios increased from 1.80 ± 1.65 (T1) to 2.98 ± 2.92 (T2), with partial attenuation at T3 (2.59 ± 2.66).

**Table 3:** Conditioned MEP ratios (mean ± SD) by ISI, group, and timepoint (fasting group; n = 30, control group; n = 11).

ISI (ms)	Group	Pre-Ramadan (T1)	Mid-Ramadan (T2)	Post-Ramadan (T3)
2	Fasting	0.68 ± 0.51	1.68 ± 2.43	1.53 ± 2.45
	Control	1.31 ± 1.17	2.50 ± 2.40	—
4	Fasting	0.82 ± 0.62	1.35 ± 1.36	1.31 ± 1.70
	Control	2.63 ± 2.83	2.90 ± 1.83	—
6	Fasting	1.19 ± 0.91	2.55 ± 3.67	1.74 ± 1.40
	Control	4.15 ± 4.41	12.76 ± 28.37^b^	—
8	Fasting	1.47 ± 1.46	3.20 ± 3.80	1.99 ± 1.70
	Control	5.12 ± 5.72	9.40 ± 15.30^b^	—
10	Fasting	1.80 ± 1.65	2.98 ± 2.92	2.59 ± 2.66
	Control	4.47 ± 4.09	9.96 ± 18.88^b^	—

MEP ratio = conditioned/ unconditioned MEP amplitude. Ratios < 1.0 = SICI; > 1.0 = ICF. — = not assessed. ^b^ High variance due to outlier influence; interpret with caution (control group n = 11).

**Table 4:** Linear mixed-effects model results (fasting group; n = 30).

Effect	df	χ²	p-value	Interpretation
Timepoint (main effect)	2	14.53	< 0.001	Longitudinal modulation of excitability confirmed
ISI (main effect)	4	107.14	< 0.001	Expected SICI-to-ICF gradient across ISIs
Timepoint × ISI (interaction)	8	6.73	0.566	Uniform global shift; no ISI-specific modulation
Group × Timepoint (exploratory)	2	4.47	0.107	Underpowered; highly exploratory

χ² = likelihood ratio test statistic. Participant as random intercept. Maximum likelihood estimation. Group × Timepoint row includes control group (T1–T2 only).

The LMM demonstrated a significant main effect of Timepoint (χ² = 14.53, df = 2, *p* < 0.001), and a significant main effect of ISI (χ² = 107.14, df = 4, *p* < 0.001). The Timepoint × ISI interaction was not significant (χ² = 6.73, df = 8, *p* = 0.566). An exploratory analysis including both groups showed a non-significant Group × Timepoint interaction (χ² = 4.47, df = 2, *p* = 0.107).

### Secondary outcomes

**Fig 4:**
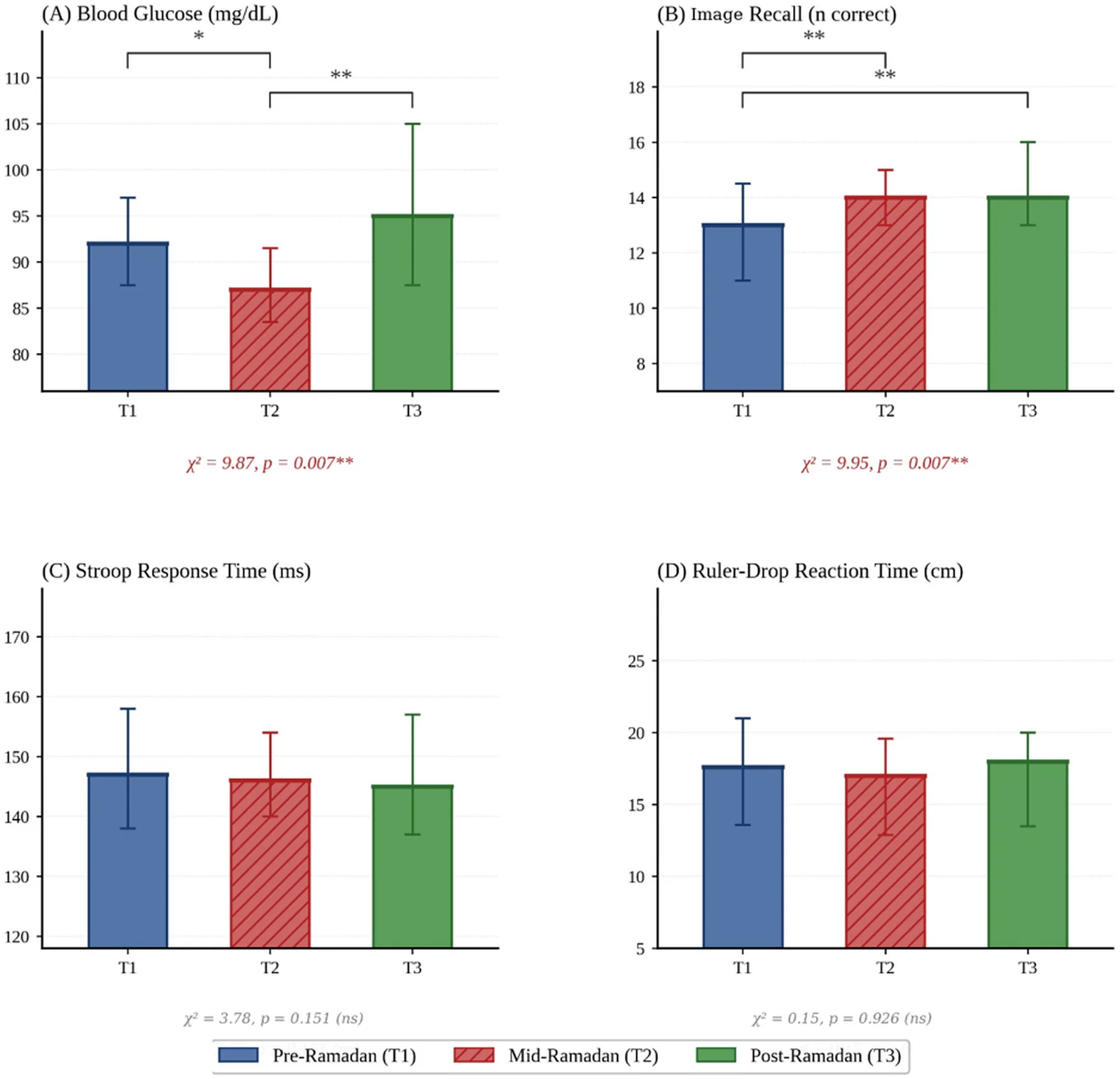
Secondary outcome measures across timepoints in the fasting group (median [IQR]). Bars show medians; error bars denote IQR. Solid blue = Pre-Ramadan (T1); red hatched = Mid-Ramadan (T2); solid green = Post-Ramadan (T3). (A) Blood glucose decreased significantly at T2 and recovered at T3 (Friedman χ² = 9.87, df = 2, p = 0.007). (B) Image recall improved significantly from T1 to T2 and T3 (Friedman χ² = 9.95, df = 2, p = 0.007). (C) Stroop RT and (D) ruler-drop RT did not change significantly (both p > 0.07). Bonferroni-corrected threshold α = 0.017.

**Fig 5:**
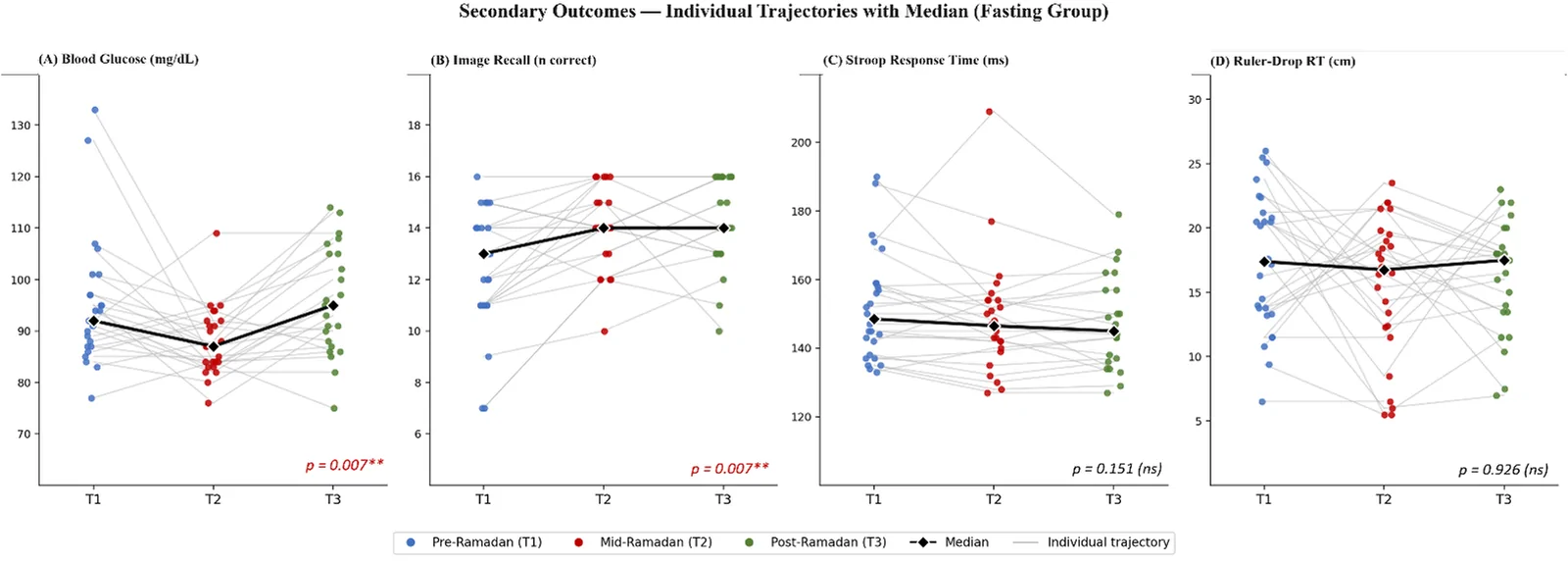
Individual trajectories of secondary outcome measures across timepoints in the fasting group. Grey lines connect each participant’s observations across T1, T2, and T3. Colored dots represent individual data points; vertical-colored bars indicate the interquartile range; the black line with diamond markers represents the group median. (A) Blood glucose (mg/dL); Friedman χ² = 9.87, *p* = 0.007. (B) Image recall (number correct); Friedman χ² = 9.95, *p* = 0.007. (C) Stroop response time (ms); *p* = 0.151 (ns). (D) Ruler-drop reaction time (cm); *p* = 0.926 (ns). Bonferroni-corrected significance threshold α = 0.017. T1 = pre-Ramadan; T2 = mid-Ramadan; T3 = post-Ramadan. ns = not significant.

Secondary outcome data for both the fasting and control groups are presented in Table 5 and Fig 4. Individual trajectories are shown in Fig 5.

**Table 5:** Secondary outcome measures by group and timepoint.

Outcome/ Group	Pre-Ramadan (T1)	Mid-Ramadan (T2)	Post-Ramadan (T3)	Friedman χ² (p)	Between-group p	Post-hoc r
**Blood glucose (mg/dL)**						
Fasting	92.0 [87.0–97.0]	86.0 [82.2–91.8]**	94.0 [86.8–105.0]	9.87 (0.007)**	T1: 0.176 T2: 0.222	T1 → T2: 0.63**; T2 → T3: 0.82**
Control	100.0 [91.2–105.5]	92.0 [83.5–97.5]	—	—		*—*
**Stroop response time (ms)**						
Fasting	150.0 [138–159]	146.5 [139–154]	146.0 [137–158]	3.78 (0.151)	T1: 0.177 T2: 0.907	*ns*
Control	139.0 [132–154]	145.0 [138–159]	—	—		*—*
**Stroop accuracy (correct/30)**						
Fasting	30.0 [30.0–30.0]	30.0 [29.0–30.0]	30.0 [30.0–30.0]	5.29 (0.071)	T1: 0.449 T2: 0.829	*ns*
Control	30.0 [29.2–30.0]	30.0 [29.0–30.0]	—	—		*—*
**Image recall (n correct)**						
Fasting	13.0 [11.0–14.0]	14.0 [13.0–15.0]**	14.0 [13.0–16.0]**	9.95 (0.007)**	T1: 0.025* T2: 0.759	T1 → T2: 0.75**; T1 → T3: 0.83**
Control	15.0 [14.0–15.0]	14.0 [12.0–15.0]	—	—		*—*
**Ruler-drop RT (cm)**						
Fasting	17.2 [13.2–20.8]	16.8 [12.7–19.7]	18.0 [13.5–20.2]	0.15 (0.926)	T1: 0.130 T2: 0.629	*ns*
Control	15.5 [10.2–17.3]	16.4 [11.5–19.0]	—	—		*—*
**Sleep duration (hrs/night)**						
Fasting	7.5 [6.5–8.5]	6.0 [5.0–6.0]**	7.0 [7.0–8.0]	27.41 (<0.001)**	T1: 0.283 T2: 0.008**	T1 → T2: 0.94**; T2 → T3: 0.98**
Control	7.0 [7.0–7.2]	7.0 [6.5–7.5]	—	—		*—*

Values are median [IQR]. Control group T3 not assessed (—). All secondary outcomes are exploratory. Within-group change across timepoints was tested with the Friedman test (χ² and p shown); between-group differences (fasting vs control at T1 and T2) with the Mann–Whitney U test; post-hoc effect sizes (r) are rank-biserial correlations from Wilcoxon signed-rank tests, fasting group only. Pairwise post-hoc comparisons (T1 vs T2, T1 vs T3, T2 vs T3) used Bonferroni-corrected α = 0.017: * p < 0.05; ** p < 0.01.

Blood glucose decreased significantly in the fasting group from T1 (median 92.0 mg/dL [IQR 87.0–97.0]) to T2 (86.0 [82.2–91.8]; W = 50, p = 0.014, r = 0.63) and rebounded to T3 (94.0 [86.8–105.0]; W = 25, p = 0.002, r = 0.82). No significant between-group differences were observed at T1 or T2 (p > 0.17).

Image recall increased significantly in the fasting group from T1 (median 13.0 [IQR 11.0–14.0]) to T2 (14.0 [13.0–15.0]; W = 34, p = 0.007, r = 0.75) and T3 (14.0 [13.0–16.0]; W = 24, p = 0.002, r = 0.83). The control group showed higher baseline values at T1 (15.0 [14.0–15.0]) than the fasting group (p = 0.025), with no difference at T2 (p = 0.759).

Stroop response time (*p* = 0.151), Stroop accuracy (*p* = 0.071), and ruler-drop reaction time (*p* = 0.926) did not change significantly in the fasting group across timepoints; these measures did not differ between groups at T1 or T2 (all *p* > 0.13).

Sleep duration decreased significantly in the fasting group from T1 (median 7.5 h [IQR 6.5–8.5]) to T2 (6.0 h [5.0–6.0]; W = 18, p < 0.001, r = 0.94) and recovered at T3 (7.0 h [7.0–8.0]; T2 vs T3: W = 5, p < 0.001, r = 0.98). Sleep duration differed between groups at T2 (p = 0.008).

## Discussion

The present study demonstrates a significant longitudinal modulation of intracortical excitability across the Ramadan period in healthy young adults observing daily intermittent fasting. Conditioned MEP ratios increased from pre-Ramadan (T1) to mid-Ramadan (T2) across all interstimulus intervals (ISIs), with partial attenuation post-Ramadan (T3). These findings indicate a time-dependent shift in intracortical excitability within the fasting group.

A significant main effect of Timepoint was observed in LMM, while the Timepoint × ISI interaction was not significant. This pattern indicates that we did not detect ISI-specific modulation. However, the absence of a statistically significant interaction should be interpreted cautiously, as the study may be underpowered to detect differential effects across ISIs. Accordingly, the results are most appropriately interpreted as evidence of a generalized change in MEP ratios over time, rather than selective modulation of inhibitory or facilitatory circuits [11,13,18]. Here, the generalized pattern — uniform across interstimulus intervals — is consistent with a systemic origin, in which cumulative sleep debt, circadian shift, and altered metabolic state act broadly rather than on a single circuit; the present design cannot, however, confirm which factor, or combination, is responsible.

Conditioned MEP ratios increased at both shorter interstimulus intervals (2–4 ms), conventionally associated with short-interval intracortical inhibition (SICI), and longer intervals (8–10 ms), associated with intracortical facilitation (ICF) [12,13,18]. Because the increase was of comparable direction across both interval ranges and the Timepoint × ISI interaction was non-significant, the pattern is best described as a uniform, group-level rise in the conditioned MEP ratio rather than as selective reduction of inhibition or selective enhancement of facilitation. Unconditioned MEP amplitude and latency remained stable across timepoints, indicating that these changes are unlikely to reflect altered corticospinal excitability and instead arise at the intracortical level [22–24].

### Intracortical excitability changes across Ramadan

The primary finding of this study — a significant, time-dependent increase in intracortical excitability peaking at mid-Ramadan — extends our understanding of how prolonged intermittent fasting influences cortical inhibition–excitation balance [8,25]. The pattern of increased MEP ratios across all ISIs at T2, with partial attenuation at T3, suggests that the neurophysiological effects of RDIF are not static but evolve dynamically across the fasting period and do not fully resolve upon its cessation. This temporal trajectory is consistent with the progressive accumulation of metabolic and circadian perturbations over the course of the month, followed by incomplete physiological recovery in the short post-Ramadan window assessed here [1,4].

RDIF is associated with metabolic and circadian alterations, including changes in glucose availability, sleep timing, and hormonal rhythms [2,4,5,26]. Circadian phase shifts have been shown to modulate cortical excitability and GABAergic inhibition, while metabolic state influences synaptic transmission and neuronal responsiveness [10,11]. These factors provide a plausible physiological context for the observed changes; however, mechanistic interpretations must remain cautious, as the present study did not directly assess neurochemical or circadian markers.

The absence of a significant Timepoint × ISI interaction should be interpreted with caution. While this null result is most appropriately interpreted as a global, ISI-nonspecific shift in excitability, the study was likely underpowered to detect differential modulation across ISIs [12,13].

### Integrated physiological context: Glucose, sleep, and cortical excitability

Blood glucose decreased at mid-Ramadan (median 92.0 to 86.0 mg/dL) and returned toward baseline post-Ramadan (94.0 mg/dL), a trajectory that matches the expected metabolic profile of daily intermittent fasting [1,8,27]. These single pre-TMS finger-prick readings are best treated as a proxy for adherence to the fasting protocol and as confirmation of a genuine shift in metabolic state, not as a measured driver of the observed changes in the conditioned MEP ratio. Fasting in this cohort did not occur in isolation: mid-Ramadan coincided with a reduction in sleep duration of approximately 1.5 hours per night and with the circadian displacement characteristic of the Ramadan schedule. Reduced glucose availability, shortened sleep, and shifted circadian timing each influence cortical excitability through partly overlapping pathways, and in this naturalistic setting they varied together rather than independently. We therefore interpret them as biologically plausible, interacting homeostatic adjustments that jointly define the mid-Ramadan physiological state, rather than as separable causes to be ranked against one another within this dataset.

Neurochemical accounts of these changes remain hypotheses that require direct testing. Glucose restriction has been linked to altered function of metabolically demanding GABAergic interneurons, and both sleep loss and circadian phase modulate GABAergic and glutamatergic signaling [9–11,28]; either could, in principle, contribute to a rise in the conditioned MEP ratio. The present study measured no neurochemical markers (plasma or cortical GABA, glutamate, BDNF, cortisol, or insulin), did not standardize time since the last meal, and was not powered to resolve interval-specific effects. Any attribution to GABA-A-mediated inhibition or glutamatergic facilitation is therefore speculative and hypothesis-generating, to be examined in adequately powered studies that pair TMS with contemporaneous metabolic, sleep, and neurochemical measurement.

### Sleep restriction as a critical confound

In fasting participants, median sleep duration decreased from 7.5 h [IQR 6.5–8.5] at T1 to 6.0 h [5.0–6.0] at T2 (a reduction of 1.5 h in the median; Friedman χ² = 27.41, *p* < 0.001; effect size *r* = 0.94 for T1–T2), returning toward baseline at T3 (Table 5). Control-group sleep was stable (7.0 h at both T1 and T2), and the between-group difference at T2 was significant (p = 0.008), which is consistent with, but does not establish, a fasting-specific reduction in sleep.

Sleep and circadian state both influence cortical excitability [10,11], and the present design cannot separate fasting-specific effects from those of reduced or shifted sleep. During Ramadan, sleep timing is typically displaced, with delayed sleep onset due to Tarawih prayers and pre-dawn Suhoor meals, resulting in reduced total sleep time and altered sleep architecture [1,4,5]. Whether a reduction of this magnitude is sufficient to alter intracortical excitability cannot be determined from the present data, and shortened sleep is therefore treated here as a plausible contributing factor rather than a quantified one.

Critically, the T2 TMS session captured participants in a state of concurrent caloric restriction, hydration deprivation, reduced sleep, and circadian phase shift — each of which independently influences cortical excitability through partially overlapping mechanisms [8,10]. This co-occurrence substantially limits causal attribution of the observed MEP ratio changes. Future studies should incorporate polysomnographic or actigraphic sleep monitoring across all timepoints, with statistical control for sleep duration and quality as covariates in the primary analysis [3,5].

### Persistence of elevated excitability at post-Ramadan

The persistence of elevated excitability at T3 may reflect residual neurophysiological adaptation following sustained metabolic perturbation [10,29], though the underlying mechanisms remain unclear. Several non-mutually exclusive explanations may be considered. First, early post-Ramadan may be insufficient for full neurophysiological recovery following 29–30 days of sustained metabolic perturbation; the cortex may undergo homeostatic plasticity changes that outlast the fasting period itself [25,29]. Second, residual sleep and dietary alterations in the early post-Ramadan period could sustain excitability elevations beyond the formal end of fasting [4,26]. Third, the partial attenuation from T2 to T3 is consistent with a gradual recovery trajectory, suggesting that a longer follow-up period — four to six weeks post-Ramadan — would be needed to establish whether excitability fully normalizes [10,24].

A specific feature of the post-Ramadan window warrants attention. The T3 assessment was deliberately scheduled after the Eid al-Fitr holiday, during the remainder of the first week following the end of Ramadan, rather than during Eid itself. This timing was intended to avoid the acute perturbations that characterize the Eid period — celebratory late-night gatherings that shorten and fragment sleep, and an abrupt dietary shift toward high simple-carbohydrate intake — which are known to influence cortical excitability [9–11]. By assessing participants after these acute festivities had subsided, the T3 measurement is less likely to reflect a transient Eid-related state. Nonetheless, the elevated MEP ratios at T3 cannot be attributed to a single process: the early post-Ramadan period may still involve incomplete recovery from 29–30 days of sustained metabolic and circadian adjustment, and residual changes in sleep and dietary patterns during this week cannot be fully excluded. The T3 findings are therefore best interpreted as reflecting a recovering rather than a fully normalized physiological state.

It should also be noted that the T3 assessment did not have a matched control group observation, precluding any between-group inference about the post-Ramadan period. Whether the persistent excitability elevation at T3 is specific to fasting individuals, or whether it reflects non-specific seasonal or habituation effects, cannot be determined from the present data [12].

### Cognitive and functional correlates

Image recall performance improved significantly in the fasting group across timepoints, with effects persisting post-Ramadan. This observation is consistent with evidence suggesting that intermittent fasting may influence cognitive function and neuroplasticity, potentially through mechanisms involving hippocampal plasticity and neurotrophic signaling pathways [29,30]. While this pattern is broadly consistent with preclinical evidence suggesting that intermittent fasting may enhance hippocampal neuroplasticity through BDNF-dependent mechanisms [25,29,30], several alternative explanations must be weighed. Practice effects warrant particular consideration. Different image sets were used at each timepoint, which limits item-specific practice effects; general task familiarity and carry-over of response strategy across sessions, however, cannot be excluded [31]. The control group showed no comparable improvement from T1 to T2 (median 15.0 → 14.0). This cohort was small and was assessed at only two timepoints, however, and this comparison is therefore highly exploratory.

The stability of Stroop response time and ruler-drop reaction time across timepoints suggests that the cognitive effects of RDIF, if present, may be domain-specific [20,21]. Alternatively, these measures may have been insufficiently sensitive to detect subtle cognitive changes in this healthy young adult sample, where ceiling and floor effects may have attenuated measurable variance [3,32].

All cognitive findings are highly exploratory and should not be interpreted as primary outcomes. Stroop accuracy was at ceiling across all timepoints (median 30/30 at T1, T2, and T3, with minimal interquartile variability), leaving insufficient variance to detect longitudinal change. The null finding for this measure is therefore uninformative and should not be interpreted as evidence that executive performance was unaffected; a more demanding executive-function task would be required to assess this domain. The stability of unconditioned MEP amplitude and latency supports the interpretation that observed ratio changes reflect intracortical rather than corticospinal mechanisms [22–24].

### Limitations and future directions

Beyond the highly exploratory status of the between-group comparisons noted above, the non-fasting cohort carries specific interpretive constraints: a small sample, high mid-Ramadan variance, the absence of a post-Ramadan (T3) assessment, and a baseline asymmetry in image-recall performance, which was higher in the non-fasting group at T1 (p = 0.025). The last of these limits any direct cross-group comparison of image-recall trajectories. The present findings are therefore best interpreted as within-subject longitudinal changes in the fasting cohort rather than as fasting-specific effects relative to non-fasting individuals.

Several additional limitations should be considered. First, potential confounding variables were not systematically controlled, including sleep duration and timing, hydration status, caffeine intake, and physical activity levels. These factors are known to influence cortical excitability and may vary substantially during Ramadan [4,5,11]. Second, although testing was conducted within a fixed afternoon window (12:00–15:00), metabolic state at the time of stimulation was not standardized, and time since last meal was not controlled. This represents a limitation, as acute metabolic conditions may directly affect TMS measures [9,28].

Third, the hormonal status of the female participants, who comprised the majority of the fasting cohort (20 of 30), was also not controlled. Fluctuations in estradiol and progesterone across the menstrual cycle modulate GABAergic neurotransmission and corticomotor excitability, including paired-pulse measures [33,34], yet menstrual-cycle phase and contraceptive use were not recorded, and assessments were timed by the Ramadan calendar rather than by cycle phase. Unmeasured variation in ovarian-hormone levels therefore represents an uncontrolled source of variance. Future studies should record cycle phase and contraceptive use and, where feasible, verify hormonal status.

Fourth, MEP ratios exhibited substantial within-group variability, particularly at longer ISIs, reflecting the inherent variability of paired-pulse TMS measures [12]. Although log transformation was applied to address non-normality, variability may still influence the precision of effect estimates. Larger sample sizes and increased trial counts per condition would improve reliability in future studies.

Finally, the sample comprised predominantly young adults, limiting generalizability to other age groups or clinical populations. The findings therefore should not be extrapolated beyond similar demographic cohorts without further study.

Future studies should include a fully matched control group assessed at all three timepoints, concurrent neurochemical sampling (plasma GABA metabolites, BDNF, cortisol, insulin), continuous actigraphic sleep monitoring, and systematic measurement of hydration and dietary intake [5,7]. Pre-specified outlier-handling protocols and adequate statistical power for interaction effects — requiring substantially larger samples than the present study — are required [12]. Extending the post-Ramadan follow-up to four to eight weeks would clarify the timescale of cortical recovery [10,29]. Investigation across diverse age groups, fasting practices, and geographical regions would also enhance the generalizability of these findings [1,2].

## Conclusion

RDIF is associated with a global, time-dependent increase in intracortical excitability, reflected by an overall increase in conditioned MEP ratios across timepoints, peaking at mid-Ramadan and partially persisting after fasting cessation. These changes occur without significant alteration of baseline corticospinal excitability and are accompanied by reductions in blood glucose and alterations in sleep duration.

Given the absence of a significant Timepoint × ISI interaction, the findings are most appropriately interpreted as a generalized shift in intracortical excitability rather than selective modulation of inhibitory or facilitatory circuits. The conditioned MEP ratio increased across both inhibition- and facilitation-associated intervals; because this interaction was not significant, however, interval-specific patterns were not statistically confirmed and are reported as exploratory.

These results indicate that the integrated metabolic, circadian, and sleep-related adaptations associated with RDIF coincide with changes in intracortical neurophysiology. Two constraints bound the conclusions that can be drawn. First, causal mechanisms remain unresolved because concurrent metabolic and sleep-related influences cannot be disentangled within the present design. Second, the non-fasting comparison cohort was assessed at pre- and mid-Ramadan only, so no matched observation exists for the post-Ramadan timepoint; the present data therefore cannot establish whether the elevated intracortical excitability observed after the end of fasting is specific to Ramadan diurnal intermittent fasting, or whether it reflects habituation to repeated paired-pulse testing or non-specific seasonal influences. All between-group comparisons and all cognitive outcomes are accordingly reported as highly exploratory, and the conclusions above rest on the within-subject longitudinal analysis of the fasting cohort. Determining the persistence and the fasting-specificity of this effect will require a fully matched cohort assessed at all three timepoints, with follow-up extended beyond the first post-Ramadan week.

## Supporting information

S1 Table STROBE Checklist. (DOCX)
