# Supplementary material for: Longitudinal changes in intracortical excitability during Ramadan diurnal intermittent fasting: A paired-pulse transcranial magnetic stimulation study
Source: PLoS One. 2026 Aug 31;21(8):e0349740. doi: 10.1371/journal.pone.0349740 (PMC13529010; doi:10.1371/journal.pone.0349740)
Supplement: Supplementary file 1 [file pone.0349740.s001.docx]

**STROBE Statement—Checklist of items that should be included in reports of cohort studies**

**Manuscript PONE-D-26-21947:** *Longitudinal Changes in Intracortical Excitability During Ramadan Diurnal Intermittent Fasting: A Paired-Pulse Transcranial Magnetic Stimulation Study*

| **Section/Topic** | **Item #** | **Recommendation** | **Location in manuscript** |
| --- | --- | --- | --- |
| **Title and abstract** | 1 | (a) Indicate the study's design with a commonly used term in the title or the abstract | Title (“longitudinal… paired-pulse TMS study”); Abstract (repeated-measures longitudinal design) |
|  |  | (b) Provide in the abstract an informative and balanced summary of what was done and what was found | Abstract |
| **Introduction** | | | |
| **Background/rationale** | 2 | Explain the scientific background and rationale for the investigation being reported | Introduction (paras 1–3) |
| **Objectives** | 3 | State specific objectives, including any prespecified hypotheses | Introduction (final paragraph – objective and hypothesis) |
| **Methods** | | | |
| **Study design** | 4 | Present key elements of study design early in the paper | Methods – Study design and timepoints; Statistical power and cohort design |
| **Setting** | 5 | Describe the setting, locations, and relevant dates, including periods of recruitment, exposure, follow-up, and data collection | Methods – Study design and timepoints (University of Sharjah, UAE; data collection 11 Feb–26 Mar 2026) |
| **Participants** | 6 | (a) Give the eligibility criteria, and the sources and methods of selection of participants. Describe methods of follow-up | Methods – Participants; Study design and timepoints |
|  |  | (b) For matched studies, give matching criteria and number of exposed and unexposed | Not applicable – non-matched observational cohort |
| **Variables** | 7 | Clearly define all outcomes, exposures, predictors, potential confounders, and effect modifiers. Give diagnostic criteria, if applicable | Methods – TMS protocol; Outcome measures |
| **Data sources/ measurement** | 8* | For each variable of interest, give sources of data and details of methods of assessment (measurement). Describe comparability of assessment methods if there is more than one group | Methods – TMS protocol; Outcome measures |
| **Bias** | 9 | Describe any efforts to address potential sources of bias | Methods – TMS protocol (fixed ISI order noted as limitation; blinding not applicable in a self-selected exposure); Statistical power and cohort design |
| **Study size** | 10 | Explain how the study size was arrived at | Methods – Statistical power and cohort design (convenience sample; no a priori power calculation) |
| **Quantitative variables** | 11 | Explain how quantitative variables were handled in the analyses. If applicable, describe which groupings were chosen and why | Methods – Statistical analysis (log-transformed MEP ratios; timepoint and ISI as factors) |
| **Statistical methods** | 12 | (a) Describe all statistical methods, including those used to control for confounding | Methods – Statistical analysis (linear mixed-effects model; Friedman, Wilcoxon, Mann-Whitney) |
|  |  | (b) Describe any methods used to examine subgroups and interactions | Methods – Statistical analysis (Timepoint × ISI interaction; exploratory Group × Timepoint) |
|  |  | (c) Explain how missing data were addressed | Methods – Statistical analysis (all available observations under a missing-at-random assumption, maximum likelihood, no imputation) |
|  |  | (d) If applicable, explain how loss to follow-up was addressed | Methods – Statistical power and cohort design (control group not assessed at T3; treated as exploratory) |
|  |  | (e) Describe any sensitivity analyses | Not applicable – no sensitivity analyses performed |
| **Results** | | | |
| **Participants** | 13* | (a) Report numbers of individuals at each stage of study | Results – Participant characteristics; Figure 1 (flowchart) |
|  |  | (b) Give reasons for non-participation at each stage | Figure 1 caption (missing/unusable MEP data at some ISIs) |
|  |  | (c) Consider use of a flow diagram | Figure 1 |
| **Descriptive data** | 14* | (a) Give characteristics of study participants and information on exposures and potential confounders | Results – Participant characteristics; Table 1 |
|  |  | (b) Indicate number of participants with missing data for each variable of interest | Figure 1 caption; Table 2 footnote |
|  |  | (c) Summarise follow-up time | Methods – Study design (T1 pre-, T2 mid-, T3 post-Ramadan) |
| **Outcome data** | 15* | Report numbers of outcome events or summary measures over time | Results; Tables 2–5; Figures 2–5 |
| **Main results** | 16 | (a) Give unadjusted estimates and their precision (eg, 95% CI). Make clear which confounders were adjusted for and why | Results (χ², p-values, rank-biserial effect sizes, medians [IQR]); Tables 3–5 |
|  |  | (b) Report category boundaries when continuous variables were categorized | Not applicable – no continuous variables categorized |
|  |  | (c) If relevant, consider translating estimates of relative risk into absolute risk | Not applicable – no risk estimates |
| **Other analyses** | 17 | Report other analyses done—eg subgroups, interactions, sensitivity analyses | Results – exploratory between-group comparisons; Statistical power and cohort design |
| **Discussion** | | | |
| **Key results** | 18 | Summarise key results with reference to study objectives | Discussion (opening paragraphs) |
| **Limitations** | 19 | Discuss limitations, taking into account sources of potential bias or imprecision. Discuss both direction and magnitude of any potential bias | Discussion – Limitations and Future Directions |
| **Interpretation** | 20 | Give a cautious overall interpretation of results considering objectives, limitations, multiplicity of analyses, and other evidence | Discussion; Conclusion |
| **Generalisability** | 21 | Discuss the generalisability (external validity) of the study results | Discussion – Limitations and Future Directions; Statistical power and cohort design |
| **Other information** | | | |
| **Funding** | 22 | Give the source of funding and the role of the funders for the present study | Funding section (no external funding received) |

**Give information separately for exposed and unexposed groups.*

*Note: An Explanation and Elaboration article discusses each checklist item and gives methodological background and published examples of transparent reporting. The STROBE checklist is best used in conjunction with this article (freely available on the Web sites of PLoS Medicine, Annals of Internal Medicine, and Epidemiology). Information on the STROBE Initiative is available at www.strobe-statement.org.*
