# Supplementary material for: Longitudinal changes in intracortical excitability during Ramadan diurnal intermittent fasting: A paired-pulse transcranial magnetic stimulation study
Source: PLoS One. 2026 Aug 31;21(8):e0349740. doi: 10.1371/journal.pone.0349740 (PMC13529010; doi:10.1371/journal.pone.0349740)
Supplement: Supplementary file 3 [file pone.0349740.s004.docx]

**Response to Reviewers**

**Manuscript PONE-D-26-21947**

*Longitudinal Changes in Intracortical Excitability During Ramadan Diurnal Intermittent Fasting: A Paired-Pulse Transcranial Magnetic Stimulation Study*

PLOS ONE — Point-by-Point Response (ordered by reviewer)

We thank the Academic Editor and all four reviewers for their careful and constructive evaluation. The comments have materially improved the manuscript. Below we respond to each reviewer in turn.

# **Reviewer 1**

**Reviewer Comment:** *The reviewer highlighted several strengths of the study, including the robust longitudinal design, use of paired-pulse TMS, inclusion of a non-fasting control group, multidimensional outcome assessment, appropriate use of linear mixed-effects modelling, conservative interpretation of the findings as a global shift in intracortical excitability, transparent acknowledgment of study limitations, and the value of the study as a foundation for future research.*

**Response:** We sincerely thank Reviewer 1 for the careful evaluation of our manuscript and for the positive assessment of our study. We appreciate the recognition of the longitudinal three-timepoint design, the use of paired-pulse TMS to assess intracortical excitability, the inclusion of a non-fasting comparison group, and the integration of neurophysiological, metabolic, sleep, and cognitive measures. We are also grateful that the reviewer considered our use of linear mixed-effects modelling and our conservative interpretation of the findings as a generalized shift in conditioned MEP ratios to be appropriate. Finally, we appreciate the acknowledgement of our transparent discussion of the study limitations and the potential of this work to inform future research. No specific revisions were requested in this review.

# **Reviewer 2**

## We note that Reviewer 2 indicated the manuscript did not meet the standard-English criterion. The full text has been revised for grammar, precision of statistical wording, and academic register.

## **Major 1 — Methodological limitations of the control group**

**Reviewer Comment:** *The control group (n = 11) is underpowered relative to the fasting group (n = 30), was not assessed at T3, showed a higher baseline Image Recall at T1 (p = 0.025), and displays extreme mid-Ramadan variance (e.g., ISI 6 ms: 12.76 ± 28.37). The authors should consider a single-arm within-subjects framing or move the control-group limitations to the forefront and label all between-group comparisons as highly exploratory.*

**Response:** We appreciate this important observation. We agree that the limitations of the non-fasting cohort should be more explicitly acknowledged. Rather than removing the control group, we retained it as an exploratory contextual comparison because it provides reference information during the pre- and mid-Ramadan assessments. We revised the manuscript to clearly define the fasting cohort as the primary longitudinal analysis and to designate all between-group comparisons and Group × Timepoint analyses as highly exploratory. We also added a dedicated subsection entitled *"Statistical power and cohort design"* and expanded the Limitations section to emphasize the small control sample, the absence of the post-Ramadan (T3) assessment, the baseline image-recall imbalance, and the high variance observed in several control-group measures.

**Changes in Manuscript:**

*New tracked insertions in the* ***Methods (Statistical power and cohort design) and Limitations and future directions sections.)***

## **Major 2 — Overstatement of SICI and ICF specificity**

**Reviewer Comment:** *The Timepoint × ISI interaction was not significant (p = 0.566), so the data strictly support a global, non-specific shift. Discussion of specific GABA-A / glutamatergic mechanisms should be substantially reduced and definitive circuit-specific claims avoided.*

**Response:** We fully agree with the reviewer and have revised the manuscript accordingly. Because the Timepoint × ISI interaction was not statistically significant, we no longer interpret the findings as evidence of selective modulation of intracortical inhibitory or facilitatory circuits. Instead, the results are consistently described as a generalized increase in conditioned MEP ratios across interstimulus intervals. We also removed statements implying preferential GABAergic impairment or disproportionate SICI reduction and substantially reduced the mechanistic discussion to avoid overinterpretation. In the Introduction, the original directional prediction of reduced SICI and enhanced ICF has been replaced with a prediction of a generalized increase in intracortical excitability, without assumption of circuit specificity. We have made this change explicit here because it alters our stated hypothesis: retaining a circuit-specific prediction while adopting the conservative, interval-nonspecific interpretation the reviewer recommends would have left the manuscript internally inconsistent. The revised wording reflects the level of inference our design can support.

**Changes in Manuscript:**

New tracked revisions in the **Introduction** and **Discussion ("Interpretation of paired-pulse TMS findings")** subsection.

## **Major 3 — The sleep confound**

**Reviewer Comment:** *Sleep in the fasting group dropped from 7.5 h (T1) to 6.0 h (T2); because participants were concurrently fasting and sleep-restricted, the TMS changes cannot be isolated to fasting. The title, abstract, and discussion should state that the changes likely reflect the combined Ramadan conditions.*

**Response:** We agree with the reviewer and revised the manuscript throughout to reflect this important distinction. The title, Abstract, Introduction, Discussion, and Conclusion now consistently describe Ramadan diurnal intermittent fasting as an integrated physiological exposure characterized by concurrent daytime fasting, reduced sleep duration, and circadian disruption. Accordingly, the observed neurophysiological changes are interpreted within this combined physiological context rather than being attributed solely to fasting. We also explicitly acknowledge that these individual components cannot be disentangled within the present naturalistic observational design.

**Changes in Manuscript:**

Tracked revisions in the **Title, Abstract, Introduction, Discussion, and Conclusion**.

## **Major 4 — Over-interpretation of cross-sectional metabolic data**

**Reviewer Comment:** *Blood glucose was a single pre-TMS finger-prick; the study lacks the continuous metabolic or neurochemical data needed for a mechanistic link. The “Metabolic Mechanisms: Glucose and GABAergic Function” section is overly speculative and should be condensed to state that glucose confirms adherence rather than serving as a proven driver.*

**Response:** We appreciate this important suggestion and substantially revised the corresponding section. The discussion now emphasizes that the single pre-TMS blood glucose measurement serves primarily as confirmation of fasting adherence and the associated metabolic state rather than evidence of a causal mechanism underlying the observed neurophysiological changes. We also reduced the mechanistic discussion and explicitly state that any proposed neurochemical explanation remains speculative because no direct neurochemical measurements were obtained in the present study.

**Changes in Manuscript:**

Tracked revisions in the **Discussion (Integrated physiological context: glucose, sleep, and cortical excitability)** subsection.

## **Minor 1 — Figure 4 legend discrepancy**

**Reviewer Comment:** *The legend states “Filled bars = T1; hatched bars = T2; open bars = T3,” which does not match the figure.*

**Response:** We thank the reviewer for identifying this inconsistency. The legend has been corrected to accurately describe the figure presentation. The revised legend now specifies the corresponding bar colors and hatching patterns for each assessment timepoint.

**Changes in Manuscript:**

Tracked revision in Figure 4.

## **Minor 2 — Table 3 footnote marker**

**Reviewer Comment:** *In Table 3, the Control-group ISI 6 ms cell shows footnote marker “t,” whereas the footnote defines “b.”*

**Response:** We thank the reviewer for this careful reading. The marker now reads "b" in all three affected Control-group cells (ISI 6, 8 and 10 ms), matching the footnote definition. We also identified that the marker had not been formatted as a superscript, which caused it to read as a stray character within the cell; all footnote markers in Tables 1 and 3, including those in the footnote definitions, are now correctly superscripted.

**Changes in Manuscript:**

Tracked revision in Table 1 and 3.

## **Minor 3 — Stroop ceiling effect**

**Reviewer Comment:** *Stroop accuracy is at an absolute ceiling (median 30.0/30.0 across timepoints); the null finding should be explicitly noted as uninformative.*

**Response:** We agree with the reviewer and have revised the Discussion to explicitly acknowledge the ceiling effect observed for Stroop accuracy in this healthy young cohort. We now clarify that the absence of significant change should be interpreted as reflecting limited task sensitivity rather than preserved executive function.

**Changes in Manuscript:**

Tracked revision in the **Discussion (Cognitive performance)** subsection.

# **Reviewer 3**

## **Point 1 — Control-group T3 absence should appear in Abstract and Conclusion**

**Reviewer Comment:** *The control group is small and had no post-Ramadan (T3) assessment, so the continuity of the fasting effect post-Ramadan cannot be confirmed. Why is this mentioned only in the Limitations? It should also be discussed in the Abstract and Conclusion.*

**Response:** We appreciate this important observation. We agree that the absence of a post-Ramadan assessment in the non-fasting cohort should be acknowledged beyond the Limitations section. Accordingly, we revised the manuscript to explicitly state this limitation in both the **Abstract** and the **Conclusion**. We also clarify that the primary findings are based on the within-subject longitudinal analysis of the fasting cohort and that, because the non-fasting group was not assessed at T3, whether the persistence of elevated intracortical excitability at T3 is specific to fasting cannot be determined.

**Changes in Manuscript:**

*Tracked revisions in the* ***Abstract and Conclusion***.

## **Point 2 — Menstrual cycle / hormonal status**

**Reviewer Comment:** *Most participants were female, yet menstrual-cycle phase, contraceptive use, and hormonal status were not addressed in the exclusion criteria; these can affect the results.*

**Response:** We thank the reviewer for this important comment, and we note that the concern was directed at the eligibility criteria specifically. We have addressed it in three places.

In the **Participants** subsection, we now state that menstrual-cycle phase, contraceptive use, and hormonal status were not applied as inclusion or exclusion criteria, as the assessment schedule was fixed by the Ramadan calendar and could not be aligned to individual cycle phase.

In **Study design and timepoints**, we describe the procedure actually followed. Female participants were not tested during menstruation, since they are exempt from fasting during that period. In three participants whose anticipated menstruation coincided with the scheduled T2 assessment, testing was instead performed on Ramadan day 12.

In **Limitations and future directions**, we state the residual constraint: although menstruation itself was avoided by rescheduling, menstrual-cycle phase outside menstruation, contraceptive use, and circulating ovarian-hormone concentrations were not systematically recorded or controlled. Because women comprised the majority of the fasting cohort (20 of 30), we identify unmeasured variation in ovarian-hormone levels as an uncontrolled source of variance, and recommend that future studies record cycle phase and contraceptive use and, where feasible, verify hormonal status.

**Changes in Manuscript:**

*New tracked insertions in the* ***Methods (Participants, and Study Design)*** *and* ***Limitations and future directions*** *sections.*

## **Point 3 — TMS procedural details (positioning, coil, hotspot)**

**Reviewer Comment:** *The methods omit participant positioning during assessment, the exact coil position, and how the motor hotspot was localized.*

**Response:** We thank the reviewer for this helpful suggestion. We have expanded the TMS protocol section to provide the missing procedural details, including participant positioning, continuous EMG monitoring to confirm muscle relaxation, coil orientation (approximately 45° to the midsagittal plane), and the systematic procedure used to localize and maintain the FDI motor hotspot throughout testing.

**Changes in Manuscript:**

New tracked insertions in the **Methods (TMS protocol)** section.

## **Point 4 — Randomization / blinding of MEP and ISI**

**Reviewer Comment:** *The authors should state whether data collection and analysis were randomized or blinded, especially for the MEP and ISI, as this would strengthen the results.*

**Response:** We thank the reviewer for this valuable suggestion. We have clarified this point in the Methods section. Because Ramadan fasting is a naturally occurring religious practice rather than an assigned intervention, group allocation was not randomized and neither participants nor investigators were blinded to fasting status. We also clarified that the interstimulus intervals were delivered in a fixed ascending order rather than a randomized or interleaved sequence and acknowledged this as a methodological limitation inherent to the naturalistic observational design.

**Changes in Manuscript:**

New tracked insertions in the **Methods (TMS protocol)** section.

# **Reviewer 4**

## **Point 1 — STROBE reporting guideline**

**Reviewer Comment:** *Did the authors follow a standardized reporting guideline (e.g., STROBE)? If so, state this in the text and provide the completed checklist as supplementary material.*

**Response:** We thank the reviewer for this valuable suggestion. We have revised the manuscript to explicitly state that this observational study was reported in accordance with the **Strengthening the Reporting of Observational Studies in Epidemiology (STROBE)** guideline. In addition, the completed STROBE checklist has been prepared and is included as Supporting Information (S1 Checklist).

**Changes in Manuscript:**

New tracked insertion in the **Methods** section. The completed STROBE checklist has been included as **Supporting Information (S1 Checklist).**.

## **Point 2 — Sample-size / power calculation**

**Reviewer Comment:** *It appears no sample-size or power calculation was performed, which is critical given the small control group. Please clarify; if recruitment was convenience-based, consider a pilot/exploratory framing.*

**Response:** We appreciate this important comment. We have substantially revised the Methods section by adding a dedicated subsection entitled **“Statistical power and cohort design.”** We now explicitly state that no *a priori* sample size calculation was performed because participant recruitment was convenience-based and constrained by the fixed duration of Ramadan, with recruitment limited to eligible participants who could be assessed within the predefined Ramadan study period. We also clarify that the fasting cohort represents the primary longitudinal analysis, whereas the between-group comparisons are exploratory.

**Changes in Manuscript:**

New tracked insertion in the Methods (Statistical power and cohort design) section.

## **Point 3 — Exact post-Ramadan (T3) day range**

**Reviewer Comment:** *The T3 assessment was said to occur “during the first week after Ramadan.” For reproducibility, please give the exact day range.*

**Response:** We thank the reviewer for this helpful suggestion. The T3 assessment was conducted within seven days of the final fasting day of Ramadan. We have revised the **Methods (Study Design)** to state this timing explicitly.

**Changes in Manuscript:**

New tracked insertions in the **Methods (Study Design and timepoints)**.

## **Point 4 — Clarification of “generalised change”**

**Reviewer Comment:** *The Discussion refers to a “generalised change in MEP ratios over time, rather than selective modulation of inhibitory or facilitatory circuits.” Did the authors mean general changes in systemic factors such as cumulative sleep debt and circadian shifts?*

**Response:** We thank the reviewer for this perceptive comment. We agree that the term *generalized* could be interpreted ambiguously. In the revised manuscript, we clarify that *generalized* refers to the statistically uniform increase in conditioned MEP ratios across all interstimulus intervals (i.e., the non-significant Timepoint × ISI interaction), rather than to selective modulation of inhibitory or facilitatory intracortical circuits. We further explain that this pattern is compatible with a broad systemic influence of Ramadan-related factors, including altered sleep, circadian rhythm, and metabolic state, while emphasizing that the present study cannot determine the relative contribution of these individual factors.

**Changes in Manuscript:**

New tracked insertion in the **Discussion.**

## **Point 5 — Eid al-Fitr context at T3**

**Reviewer Comment:** *In the “Persistence of Elevated Excitability at Post-Ramadan” subsection, integrate the cultural/physiological context of Eid al-Fitr, since the days after Ramadan involve sleep disruption and rapid dietary change. Discuss whether the holiday acts as an independent confounder at T3 or is part of gradual recovery.*

**Response:** We thank the reviewer for this thoughtful comment. We have expanded the Discussion to acknowledge that the T3 assessment was performed after the Eid al-Fitr holiday and to discuss the potential influence of post-Ramadan behavioral changes on the findings. We explain that, although an acute Eid-related effect is less likely, the early post-Ramadan period may still represent a recovering physiological state. We therefore acknowledge that residual changes in sleep, diet, and circadian rhythm during this period cannot be fully separated from recovery following Ramadan fasting, and the T3 findings should be interpreted within this combined physiological context.

**Changes in Manuscript:**

New tracked insertion in the **Discussion ("Persistence of Elevated Excitability at Post-Ramadan")** subsection.

# **Academic Editor & Journal Requirements**

## **Ethics-statement placement (Journal Requirement 2)**

**Reviewer Comment:** *The ethics statement must appear only in the Methods section.*

**Response:** Corrected. The ethics statement is retained within the Methods (“Ethics statement” subsection) and the duplicate standalone “Ethics Statement” section near the end of the manuscript has been removed. The Declaration of Helsinki statement was consolidated into the Methods ethics statement.

We again thank the Academic Editor and the reviewers for their constructive comments and valuable suggestions. We believe that the revisions have substantially strengthened the manuscript, and we hope that the revised version is now suitable for publication in *PLOS ONE*.
