# Supplementary material for: Longitudinal changes in intracortical excitability during Ramadan diurnal intermittent fasting: A paired-pulse transcranial magnetic stimulation study
Source: PLoS One. 2026 Aug 31;21(8):e0349740. doi: 10.1371/journal.pone.0349740 (PMC13529010; doi:10.1371/journal.pone.0349740)
Supplement: Supplementary file 4 [file pone.0349740.s002.docx]

Here is a formal, structured peer-review letter formatted for direct submission to the journal. It maintains a professional, constructive, and objective academic tone.

**Reviewer Comments to Authors**

**Manuscript Number:** PONE-D-26-21947

**Title:** Longitudinal Changes in Intracortical Excitability During Ramadan Fasting: A Paired-Pulse Transcranial Magnetic Stimulation Study

**Overall Assessment**

The authors present an interesting longitudinal study investigating the effects of prolonged daily intermittent fasting (Ramadan) on intracortical excitability using paired-pulse transcranial magnetic stimulation (TMS). The study’s longitudinal design across pre-, mid-, and post-Ramadan timepoints is a notable strength, addressing a gap in the literature regarding dynamic neurophysiological shifts in naturalistic fasting models. The primary finding of a significant main effect of timepoint on conditioned MEP ratios is intriguing.

However, there are substantial methodological and interpretive limitations—particularly concerning the control group, confounding variables, and the overstatement of specific mechanistic pathways—that must be addressed before the manuscript can be considered suitable for publication.

**Major Revisions Required**

**1. Methodological Inadequacies of the Control Group**

The inclusion of the control group currently weakens the manuscript's conclusions due to significant disparities and missing data:

- **Sample Size Imbalance:** The control group ($n=11$) is severely underpowered compared to the fasting group ($n=30$), limiting the validity of between-group comparisons.
- **Missing Timepoints:** The control group was not assessed at the post-Ramadan timepoint (T3). Consequently, it is impossible to determine if the persistent excitability at T3 in the fasting group reflects a residual fasting effect, a habituation to the TMS protocol, or a non-specific seasonal effect.
- **Baseline Asymmetries:** The control group exhibited significantly higher baseline scores in the Image Recall task at T1 ($p=0.025$), indicating a lack of baseline equivalence.
- **Extreme Variance:** As noted in Table 3, the control group’s mid-Ramadan (T2) MEP ratios display extreme variance (e.g., ISI 6 ms: 12.76 ± 28.37) driven by outliers, further compromising the reliability of this dataset.
- *Recommendation:* The authors should strongly consider framing this manuscript as a single-arm, within-subjects observational study focused exclusively on the fasting cohort. If the control group is retained, its limitations must be moved from the end of the discussion to the forefront, and all between-group statistical comparisons should be explicitly labeled as highly exploratory or removed entirely.

**2. Overstatement of SICI and ICF Specificity**

The manuscript's language frequently implies specific modulation of short-interval intracortical inhibition (SICI) and intracortical facilitation (ICF) circuits.

- While the Linear Mixed Model (LMM) revealed a significant main effect of Timepoint, the Timepoint × ISI interaction was not statistically significant ($p=0.566$).
- *Recommendation:* The data strictly supports a *global*, non-specific shift in excitability. The discussion surrounding specific GABA-A and glutamatergic mechanisms must be heavily truncated. The authors should adhere to the conservative interpretation that fasting induced a generalized increase in the MEP ratio across all intervals, avoiding definitive claims about specific inhibitory or facilitatory circuit modulation.

**3. The Sleep Confound**

The study identifies a major confounding variable that threatens the primary hypothesis: acute sleep restriction.

- Sleep duration in the fasting group dropped significantly from 7.5 hours at T1 to 6.0 hours at T2. The authors correctly acknowledge that sleep restriction independently modulates cortical excitability.
- *Recommendation:* Because participants were concurrently fasting and sleep-restricted, it is impossible to isolate the TMS changes specifically to the metabolic effects of fasting. The title, abstract, and discussion must more explicitly state that the observed neurophysiological changes likely result from the *combined* conditions of Ramadan (fasting, circadian shifts, and sleep restriction), rather than attributing the changes solely to the fasting-related metabolic state.

**4. Over-Interpretation of Cross-Sectional Metabolic Data**

The authors attempt to mechanistically link the changes in cortical excitability to metabolic shifts, specifically citing reduced blood glucose.

- Blood glucose was assessed via a single finger-prick prior to TMS. The study lacks the continuous metabolic or neurochemical data required to establish this link.
- *Recommendation:* The section titled "Metabolic Mechanisms: Glucose and GABAergic Function" is overly speculative. This section should be condensed to emphasize that the glucose readings simply confirm adherence to the fasting protocol, rather than serving as a proven mechanistic driver for the observed TMS results.

**Minor Revisions & Technical Corrections**

- **Figure 4 Legend Discrepancy:** The figure legend for Figure 4 states "Filled bars = Pre-Ramadan (T1); hatched bars = Mid-Ramadan (T2); open bars = Post-Ramadan (T3)". However, the visual representations in Figure 4 (A-D) appear to utilize solid colored bars rather than hatched or open styling. Please update the legend to accurately match the visual output.
- **Table 3 Typographical Error:** In Table 3, the row for the Control Group at ISI 6 ms contains a typographical error in the footnote marker. It reads "$12.76 \pm 28.37~t$", whereas the corresponding footnote utilizes a "$b$".
- **Cognitive Task Floor/Ceiling Effects:** Stroop accuracy appears to be at an absolute ceiling, with medians of 30.0/30.0 across all timepoints. While the authors briefly note potential ceiling effects, they should explicitly state that this specific task was insufficiently challenging for this healthy cohort to yield meaningful variance, rendering the null findings for this specific metric uninformative.
