# Supplementary material for: Longitudinal changes in intracortical excitability during Ramadan diurnal intermittent fasting: A paired-pulse transcranial magnetic stimulation study
Source: PLoS One. 2026 Aug 31;21(8):e0349740. doi: 10.1371/journal.pone.0349740 (PMC13529010; doi:10.1371/journal.pone.0349740)
Supplement: Peer review history

# Peer review: Decision Letter 0

**DOI:** 10.1371/journal.pone.0349740.r001

**Authors:** MoezAlIslam Faris

2 Jun 2026

Dear Dr. Kim,

Thank you for submitting your manuscript to PLOS ONE. After careful consideration, we feel that it has merit but does not fully meet PLOS ONE’s publication criteria as it currently stands. Therefore, we invite you to submit a revised version of the manuscript that addresses the points raised during the review process.

Please submit your revised manuscript by Aug 01 2026 11:59PM. If you will need more time than this to complete your revisions, please reply to this message or contact the journal office at plosone@plos.org. When you're ready to submit your revision, log on to https://www.editorialmanager.com/pone/ and select the 'Submissions Needing Revision' folder to locate your manuscript file.

- A letter that responds to each point raised by the academic editor and reviewer(s). You should upload this letter as a separate file labeled 'Response to Reviewers'.
- A marked-up copy of your manuscript that highlights changes made to the original version. You should upload this as a separate file labeled 'Revised Manuscript with Track Changes'.
- An unmarked version of your revised paper without tracked changes. You should upload this as a separate file labeled 'Manuscript'.

If you would like to make changes to your financial disclosure, please include your updated statement in your cover letter. Guidelines for resubmitting your figure files are available below the reviewer comments at the end of this letter.

If applicable, we recommend that you deposit your laboratory protocols in protocols.io to enhance the reproducibility of your results. Protocols.io assigns your protocol its own identifier (DOI) so that it can be cited independently in the future. For instructions see: https://journals.plos.org/plosone/s/submission-guidelines#loc-laboratory-protocols. Additionally, PLOS ONE offers an option for publishing peer-reviewed Lab Protocol articles, which describe protocols hosted on protocols.io. Read more information on sharing protocols at https://plos.org/protocols?utm_medium=editorial-email&utm_source=authorletters&utm_campaign=protocols.

As the corresponding author, your ORCID iD is verified in the submission system and will appear in the published article. PLOS supports the use of ORCID, and we encourage all coauthors to register for an ORCID iD and use it as well. Please encourage your coauthors to verify their ORCID iD within the submission system before final acceptance, as unverified ORCID iDs will not appear in the published article. *Only*  the individual author can complete the verification step; PLOS staff *cannot*  verify ORCID iDs on behalf of authors.

We look forward to receiving your revised manuscript.

Kind regards,

MoezAlIslam Faris, PhD

Academic Editor

PLOS One

**Journal Requirements:**

1. When submitting your revision, we need you to address these additional requirements.

Please ensure that your manuscript meets PLOS ONE's style requirements, including those for file naming. The PLOS ONE style templates can be found at

https://journals.plos.org/plosone/s/file?id=wjVg/PLOSOne_formatting_sample_main_body.pdf and

https://journals.plos.org/plosone/s/file?id=ba62/PLOSOne_formatting_sample_title_authors_affiliations.pdf

2. Your ethics statement should only appear in the Methods section of your manuscript. If your ethics statement is written in any section besides the Methods, please delete it from any other section.

3. If the reviewer comments include a recommendation to cite specific previously published works, please review and evaluate these publications to determine whether they are relevant and should be cited. There is no requirement to cite these works unless the editor has indicated otherwise. 

[Note: HTML markup is below. Please do not edit.]

Reviewers' comments:

**Comments to the Author**

1. Is the manuscript technically sound, and do the data support the conclusions?

Reviewer #1: Yes

Reviewer #2: Yes

Reviewer #3: Partly

Reviewer #4: Yes

**********

2. Has the statistical analysis been performed appropriately and rigorously?

Reviewer #1: Yes

Reviewer #2: Yes

Reviewer #3: Yes

Reviewer #4: Yes

**********

3. Have the authors made all data underlying the findings in their manuscript fully available?

The PLOS Data policy

Reviewer #1: Yes

Reviewer #2: Yes

Reviewer #3: Yes

Reviewer #4: Yes

**********

4. Is the manuscript presented in an intelligible fashion and written in standard English?

Reviewer #1: Yes

Reviewer #2: No

Reviewer #3: Yes

Reviewer #4: Yes

**********

**Reviewer #1:**  During review it came clear the positive following point of the study:

1. Robust Methodological Design Advanced Neurophysiological Technique: Utilizing paired-pulse Transcranial Magnetic Stimulation (TMS) provides a highly precise, non-invasive probe of human intracortical circuitry. Longitudinal Tracking: Assessing participants across three distinct timepoints (pre-, mid-, and post-Ramadan) successfully captures the dynamic, time-dependent trajectory of neurological adaptations. Control Group Integration: Including a non-fasting control group strengthens the internal validity by ruling out confounding time-dependent variables or repetition effects.

2. Comprehensive Outlines & Holistic Approach Multidimensional Outcomes: Integrating secondary measures like blood glucose, sleep duration, and cognitive performance bridges the gap between neurophysiology, metabolism, and behavior. Naturalistic Model: Framing Ramadan as a natural model for prolonged daily intermittent fasting offers highly ecologically valid insights into real-world human physiology.

3. Statistical Rigor and Objective Interpretation Sophisticated Analytics: Applying a linear mixed-effects model is the optimal statistical choice for longitudinal data with missing values or repeated measures. Balanced Concept Interpretation: Characterizing the findings as a "global shift" in excitability—rather than over-interpreting specific inhibitory or facilitatory circuit alterations—demonstrates high scientific integrity.

4. Scientific Transparency Honest Limitation Acknowledgment: Explicitly stating that metabolic states and sleep deprivation cannot be fully disentangled within this design highlights strong academic transparency. Foundation for Future Research: This clear limitation provides a direct, actionable roadmap for future studies looking to isolate these overlapping physiological variables.

**Reviewer #2:**  Here is a formal, structured peer-review letter formatted for direct submission to the journal. It maintains a professional, constructive, and objective academic tone.

---

**Reviewer Comments to Authors**

**Manuscript Number:** PONE-D-26-21947

**Title:** Longitudinal Changes in Intracortical Excitability During Ramadan Fasting: A Paired-Pulse Transcranial Magnetic Stimulation Study

**Overall Assessment**

The authors present an interesting longitudinal study investigating the effects of prolonged daily intermittent fasting (Ramadan) on intracortical excitability using paired-pulse transcranial magnetic stimulation (TMS). The study’s longitudinal design across pre-, mid-, and post-Ramadan timepoints is a notable strength, addressing a gap in the literature regarding dynamic neurophysiological shifts in naturalistic fasting models. The primary finding of a significant main effect of timepoint on conditioned MEP ratios is intriguing.

However, there are substantial methodological and interpretive limitations—particularly concerning the control group, confounding variables, and the overstatement of specific mechanistic pathways—that must be addressed before the manuscript can be considered suitable for publication.

---

**Major Revisions Required**

**1. Methodological Inadequacies of the Control Group**

The inclusion of the control group currently weakens the manuscript's conclusions due to significant disparities and missing data:

* **Sample Size Imbalance:** The control group ($n=11$) is severely underpowered compared to the fasting group ($n=30$), limiting the validity of between-group comparisons.

* **Missing Timepoints:** The control group was not assessed at the post-Ramadan timepoint (T3). Consequently, it is impossible to determine if the persistent excitability at T3 in the fasting group reflects a residual fasting effect, a habituation to the TMS protocol, or a non-specific seasonal effect.

* **Baseline Asymmetries:** The control group exhibited significantly higher baseline scores in the Image Recall task at T1 ($p=0.025$), indicating a lack of baseline equivalence.

* **Extreme Variance:** As noted in Table 3, the control group’s mid-Ramadan (T2) MEP ratios display extreme variance (e.g., ISI 6 ms: 12.76 ± 28.37) driven by outliers, further compromising the reliability of this dataset.

* *Recommendation:* The authors should strongly consider framing this manuscript as a single-arm, within-subjects observational study focused exclusively on the fasting cohort. If the control group is retained, its limitations must be moved from the end of the discussion to the forefront, and all between-group statistical comparisons should be explicitly labeled as highly exploratory or removed entirely.

**2. Overstatement of SICI and ICF Specificity**

The manuscript's language frequently implies specific modulation of short-interval intracortical inhibition (SICI) and intracortical facilitation (ICF) circuits.

* While the Linear Mixed Model (LMM) revealed a significant main effect of Timepoint, the Timepoint × ISI interaction was not statistically significant ($p=0.566$).

* *Recommendation:* The data strictly supports a *global*, non-specific shift in excitability. The discussion surrounding specific GABA-A and glutamatergic mechanisms must be heavily truncated. The authors should adhere to the conservative interpretation that fasting induced a generalized increase in the MEP ratio across all intervals, avoiding definitive claims about specific inhibitory or facilitatory circuit modulation.

**3. The Sleep Confound**

The study identifies a major confounding variable that threatens the primary hypothesis: acute sleep restriction.

* Sleep duration in the fasting group dropped significantly from 7.5 hours at T1 to 6.0 hours at T2. The authors correctly acknowledge that sleep restriction independently modulates cortical excitability.

* *Recommendation:* Because participants were concurrently fasting and sleep-restricted, it is impossible to isolate the TMS changes specifically to the metabolic effects of fasting. The title, abstract, and discussion must more explicitly state that the observed neurophysiological changes likely result from the *combined* conditions of Ramadan (fasting, circadian shifts, and sleep restriction), rather than attributing the changes solely to the fasting-related metabolic state.

**4. Over-Interpretation of Cross-Sectional Metabolic Data**

The authors attempt to mechanistically link the changes in cortical excitability to metabolic shifts, specifically citing reduced blood glucose.

* Blood glucose was assessed via a single finger-prick prior to TMS. The study lacks the continuous metabolic or neurochemical data required to establish this link.

* *Recommendation:* The section titled "Metabolic Mechanisms: Glucose and GABAergic Function" is overly speculative. This section should be condensed to emphasize that the glucose readings simply confirm adherence to the fasting protocol, rather than serving as a proven mechanistic driver for the observed TMS results.

---

**Minor Revisions & Technical Corrections**

* **Figure 4 Legend Discrepancy:** The figure legend for Figure 4 states "Filled bars = Pre-Ramadan (T1); hatched bars = Mid-Ramadan (T2); open bars = Post-Ramadan (T3)". However, the visual representations in Figure 4 (A-D) appear to utilize solid colored bars rather than hatched or open styling. Please update the legend to accurately match the visual output.

* **Table 3 Typographical Error:** In Table 3, the row for the Control Group at ISI 6 ms contains a typographical error in the footnote marker. It reads "$12.76 \pm 28.37~t$", whereas the corresponding footnote utilizes a "$b$".

* **Cognitive Task Floor/Ceiling Effects:** Stroop accuracy appears to be at an absolute ceiling, with medians of 30.0/30.0 across all timepoints. While the authors briefly note potential ceiling effects, they should explicitly state that this specific task was insufficiently challenging for this healthy cohort to yield meaningful variance, rendering the null findings for this specific metric uninformative.

**Reviewer #3:**  The research is considered one of the interesting topics in connection between the nutrition field and physical therapy science. But the research requires some revisions according to the following points :

1-You mensioned in the limitations that the control group is small and you did not get any data or assessment from this group post Ramadan as T3 in the fasting group (How we can approve the continuity of the Effect of fasting post Ramadan?!).

I need explanation and why you mensioned this only in the limitations? it should be discussed in the abstract and the conclusion.

2-Although Most of the cases are females,but there is no mention of the menstrual cycle, contraceptive pills or hormonal status in the exclusion criteria.

All of these can affect the results

3-In methodology the authors mentioned most of the procedures except the position of the paient during assesment, the coil accurate position and the motor hotspot localization How did you detect it?

4-the authors should mension if the data collection and analysis was randomized or blinded in the research Especially the MEP and ISI, this will strength the results.

Finally thanks and I hope that i could help in releasing a new topic that deserves appreciation.

**Reviewer #4:**  General Comment

1- Did the authors follow a standardised reporting guideline for this manuscript (e.g., the STROBE statement )? If so, please state this clearly in the text and provide the completed checklist as supplementary material.

Methodology Section

2- It seems that a sample size or statistical power calculation does was not performed, which might be critical due to the small size of the control group. Please clarify whether such a power calculation was done. If recruitment was based on convenience sampling without a power analysis, the authors should consider reframing this manuscript as a pilot or exploratory study.

3- Regarding the post-Ramadan (T3) assessment, it was mentioned that it was done during the first week after Ramadan. To ensure study reproducibility, please mention in the exact day range (e.g., 24 hours, 48 hours, etc…).

Discussion Section

4- In the discussion section the authors mentioned "generalised change in MEP ratios over time, rather than selective modulation of inhibitory or facilitatory circuits". Did the authors mean the general changes in systemic factors such as cumulative sleep debt and circadian rhythm shifts?

5- In the subsection "Persistence of Elevated Excitability at Post-Ramadan", the authors should integrate the cultural and physiological context of the Eid al-Fitr holiday, as the days following Ramadan are typically causes sleep disruptions and rapid dietary changes which are often caused by increased intake of simple carbohydrate foods.

In other words, I think it would increase the depth of the discussion if the authors explain more in depth if the holiday phase acts as an independent confounding variable on cortical excitability at timepoint 3 or is it is considered a component of a gradual neurophysiological recovery from the Ramadan fasting.

**********

what does this mean?). If published, this will include your full peer review and any attached files.

If you choose “no”, your identity will remain anonymous but your review may still be made public.

**Do you want your identity to be public for this peer review?**  For information about this choice, including consent withdrawal, please see our Privacy Policy

Reviewer #1: No

Reviewer #2: No

Reviewer #3: No

Reviewer #4: **Yes:**  Wesam A. Debes

**********

[NOTE: If reviewer comments were submitted as an attachment file, they will be attached to this email and accessible via the submission site. Please log into your account, locate the manuscript record, and check for the action link "View Attachments". If this link does not appear, there are no attachment files.]

To ensure your figures meet our technical requirements, please review our figure guidelines: https://journals.plos.org/plosone/s/figures

You may also use PLOS’s free figure tool, NAAS, to help you prepare publication quality figures: https://journals.plos.org/plosone/s/figures#loc-tools-for-figure-preparation.

NAAS will assess whether your figures meet our technical requirements by comparing each figure against our figure specifications.

Attachment Submitted filename: PLOS-PONE-D-26-21947.docx

# Author comment: Author response to Decision Letter 1

**DOI:** 10.1371/journal.pone.0349740.r002

30 Jul 2026

We thank the Academic Editor and all reviewers for their thoughtful and constructive comments. We have carefully revised the manuscript in response to all comments. A detailed point-by-point response is provided in the attached "Response to Reviewers" document. We have also submitted both a tracked-changes version and a clean version of the revised manuscript.

Attachment Submitted filename: Response_to_Reviewers_PONE-D-26-21947.docx

# Editor report: Decision Letter 1

**DOI:** 10.1371/journal.pone.0349740.r003

**Authors:** MoezAlIslam Faris

16 Aug 2026

Longitudinal Changes in Intracortical Excitability During Ramadan Diurnal Intermittent Fasting: A Paired-Pulse Transcranial Magnetic Stimulation Study

PONE-D-26-21947R1

Dear Dr. Kim,

We’re pleased to inform you that your manuscript has been judged scientifically suitable for publication and will be formally accepted for publication once it meets all outstanding technical requirements.

Within one week, you’ll receive an e-mail detailing the required amendments. When these have been addressed, you’ll receive a formal acceptance letter and your manuscript will be scheduled for publication.

An invoice will be generated when your article is formally accepted. Please note, if your institution has a publishing partnership with PLOS and your article meets the relevant criteria, all or part of your publication costs will be covered. Please make sure your user information is up-to-date by logging into Editorial Manager at Editorial Manager® and clicking the ‘Update My Information' link at the top of the page. For questions related to billing, please contact billing support.

If your institution or institutions have a press office, please notify them about your upcoming paper to help maximize its impact. If they’ll be preparing press materials, please inform our press team as soon as possible -- no later than 48 hours after receiving the formal acceptance. Your manuscript will remain under strict press embargo until 2 pm Eastern Time on the date of publication. For more information, please contact onepress@plos.org.

Kind regards,

MoezAlIslam Faris, PhD

Academic Editor

PLOS One

Additional Editor Comments (optional):

Reviewers' comments:

# Editor report: Acceptance letter

**DOI:** 10.1371/journal.pone.0349740.r004

**Authors:** MoezAlIslam Faris

PONE-D-26-21947R1

PLOS One

Dear Dr. Kim,

I'm pleased to inform you that your manuscript has been deemed suitable for publication in PLOS One. Congratulations! Your manuscript is now being handed over to our production team.

At this stage, our production department will prepare your paper for publication. This includes ensuring the following:

* All references, tables, and figures are properly cited

* All relevant supporting information is included in the manuscript submission,

* There are no issues that prevent the paper from being properly typeset

You will receive further instructions from the production team, including instructions on how to review your proof when it is ready. Please keep in mind that we are working through a large volume of accepted articles, so please give us a few days to review your paper and let you know the next and final steps.

Lastly, if your institution or institutions have a press office, please let them know about your upcoming paper now to help maximize its impact. If they'll be preparing press materials, please inform our press team within the next 48 hours. Your manuscript will remain under strict press embargo until 2 pm Eastern Time on the date of publication. For more information, please contact onepress@plos.org.

You will receive an invoice from PLOS for your publication fee after your manuscript has reached the completed accept phase. If you receive an email requesting payment before acceptance or for any other service, this may be a phishing scheme. Learn how to identify phishing emails and protect your accounts at https://explore.plos.org/phishing.

If we can help with anything else, please email us at customercare@plos.org.

Thank you for submitting your work to PLOS One and supporting open access.

Kind regards,

PLOS ONE Editorial Office Staff

on behalf of

Professor MoezAlIslam Faris

Academic Editor

PLOS One
